# Neuroticism explains unwanted variance in Implicit Association Tests of personality: possible evidence for an affective valence confound

**DOI:** 10.3389/fpsyg.2013.00672

**Published:** 2013-09-30

**Authors:** Monika Fleischhauer, Sören Enge, Robert Miller, Alexander Strobel, Anja Strobel

**Affiliations:** Department of Psychology, Technische Universitaet DresdenDresden, Germany

**Keywords:** Implicit Association Test, unwanted variance, method variance, personality, neuroticism

## Abstract

Meta-analytic data highlight the value of the Implicit Association Test (IAT) as an indirect measure of personality. Based on evidence suggesting that confounding factors such as cognitive abilities contribute to the IAT effect, this study provides a first investigation of whether basic personality traits explain unwanted variance in the IAT. In a gender-balanced sample of 204 volunteers, the Big-Five dimensions were assessed via self-report, peer-report, and IAT. By means of structural equation modeling (SEM), latent Big-Five personality factors (based on self- and peer-report) were estimated and their predictive value for unwanted variance in the IAT was examined. In a first analysis, unwanted variance was defined in the sense of method-specific variance which may result from differences in task demands between the two IAT block conditions and which can be mirrored by the absolute size of the IAT effects. In a second analysis, unwanted variance was examined in a broader sense defined as those systematic variance components in the raw IAT scores that are not explained by the latent implicit personality factors. In contrast to the absolute IAT scores, this also considers biases associated with the direction of IAT effects (i.e., whether they are positive or negative in sign), biases that might result, for example, from the IAT's stimulus or category features. None of the explicit Big-Five factors was predictive for method-specific variance in the IATs (first analysis). However, when considering unwanted variance that goes beyond pure method-specific variance (second analysis), a substantial effect of neuroticism occurred that may have been driven by the affective valence of IAT attribute categories and the facilitated processing of negative stimuli, typically associated with neuroticism. The findings thus point to the necessity of using attribute category labels and stimuli of similar affective valence in personality IATs to avoid confounding due to recoding.

## Introduction

During the last decade, much attention has been focused on *indirect measures* of personality as it has been shown that personality will be better understood if both explicit and implicit aspects of a construct are considered. Specifically, as condensed in the Reflective-Impulsive Model (Strack and Deutsch, 2004) and the Behavioral Process Model of Personality (Back et al., 2009), human behavior can be conceptualized as a function of two distinct systems: First, a *reflective system* is supposed that elicits behavior as a consequence of deliberated decision-processes leading to explicit memory representations that can be best measured via self-report questionnaires. Second, a fast acting *impulsive system* is assumed that activates behavioral schemata by spread-of-activation processes without the need of individual's intention. Such processes are assumed to be accumulated as implicit memory representations that can best be accessed via indirect measures. In fact, indirect measures of personality have been shown to provide incremental validity over and above self-reports predicting the more involuntary automatic aspects of personality-related behavior (for an overview, see Greenwald et al., 2009; see also Asendorpf et al., 2002; Perugini, 2005; Back et al., 2009; Fleischhauer et al., 2013; but for a controversial discussion of the Implicit Association Test (IAT's) predictive validity, see Oswald et al., 2013).

**Figure 1:**
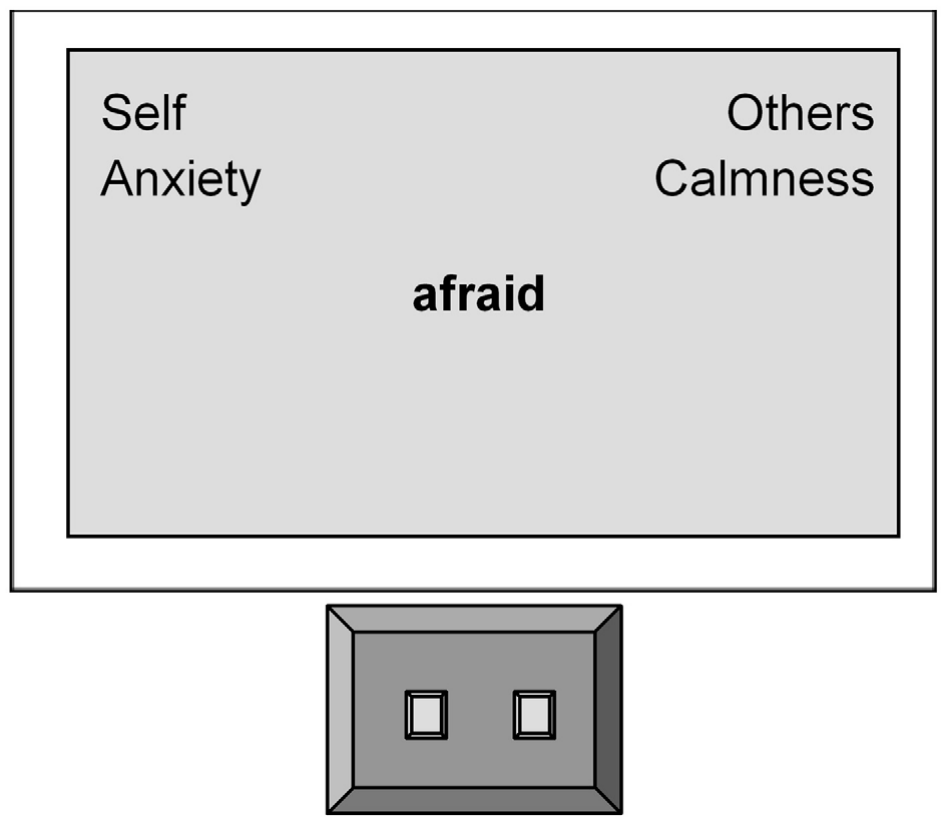
**Illustration of the Implicit Association Test with the target categories “Self” and “Others” and the attribute categories “Anxiety” and “Calmness” to which stimuli (e.g., afraid) must be categorized by using two response keys**. Note that the position (left, right) of the attribute categories is changed within the paradigm.

In particular, the IAT introduced by Greenwald et al. (1998) has attracted considerable attention. The IAT measures the relative strength of associations between bipolar target categories (for personality IATs, typically the categories *Self* vs. *Others*) and bipolar attribute categories (for trait anxiety, e.g., *Anxiety* vs. *Calmness*). Individual exemplars of the categories are presented in the center of a computer screen and participants are instructed to sort these items according to their category membership. Although the items must be assigned to four different categories, participants have only two response keys to do so. Consequently, each target category shares the response key with one attribute category; and key assignment of the attribute categories is changed within the IAT procedure (for an illustration, see Figure 1). Typically, the IAT contains five blocks: In block 1, participants practice the response key assignment of the two target categories (target discrimination). With respect to the anxiety IAT outlined above, for example, individuals must press the left key for items of the category “Self” and the right key for items of the category “Others.” In block 2, attribute key assignment is practiced (attribute discrimination) with left responses for items of the category “Anxiety” and right responses for exemplars of “Calmness.” In block 3, both target- and attribute discrimination is combined. In block 4, then, the key assignment of the attribute categories is changed and individuals now must press the left key for items of the category “Calmness” and the right key for exemplars of “Anxiety.” Again, in block 5, target and attribute discrimination tasks are combined. However, individuals must respond with the left key to exemplars of “Self” and “Calmness” and with the right key to exemplars of “Others” and Anxiety.” In standard IAT procedures, stimuli of the target- and attribute categories are presented in alternating order, that is, individuals must switch between attribute and target discrimination for each trial.

It is assumed that an individual's response is faster when two strongly associated categories share one response key (individual's compatible block, for a highly anxious individual this would be the block where *Self* is combined with *Anxiety*) than when these concepts share different keys (individual's incompatible block). The *IAT effect* as the outcome measure of the IAT then reflects the performance difference between the two category combinations.

Despite the promising results regarding reliability and predictive validity (see e.g., Schnabel et al., 2008), a growing body of research indicates that the IAT effect might also contain construct-unrelated variance, mainly resulting from the IAT's block design and the compatibility of category combination. More precisely, compatibility has been found to influence speed-accuracy settings with slower and more accurate responses (conservative response criterion) in the incompatible block and fast and less accurate responses (liberal response criterion) in the compatible block (Brendl et al., 2001; Klauer et al., 2007). Furthermore, it has been noted that the two combined IAT blocks asymmetrically allow for the use of recoding strategies (see De Houwer, 2001; Mierke and Klauer, 2001, 2003; Rothermund and Wentura, 2001, 2004). For a personality IAT measuring anxiety, Schnabel et al. (2006) found evidence for recoding based on the affective valence (negative vs. positive) of the attribute concepts instead of their semantic meaning (anxious vs. self-confident) as intended. Because individuals may typically have a rather positive self-concept, items can be categorized in terms of the single feature dichotomy “positive” vs. “negative” in the IAT block, where the more positive attribute concept “self-confident” is combined with the target category “Me” (and the negatively valenced category “anxious” with “Others”). Because of the common feature “positive” vs. “negative” in this block, individuals do not have to switch between the target- and the attribute-categorization rule when categorizing stimuli (Mierke and Klauer, 2001). In the IAT block combining the more negative attribute “anxious” with “Me,” however, recoding is impossible and participants must switch between the two discrimination tasks (i.e., sorting items to the target vs. the attribute concepts).

Taking into account that the two combined blocks require different demands on cognitive control, it appears obvious that individual differences in executive functions may systematically influence the IAT effect. The more cognitive skills will be applied during the more demanding IAT block, the smaller the IAT effect will be (see McFarland and Crouch, 2002; Klauer et al., 2007; Sherman et al., 2008). This demonstrates that the IAT's block design can cause unwanted variance in the IAT effect (so called method-specific variance) that potentially impairs the IAT's validity. In a recent study, Klauer et al. (2010) examined whether the three higher-order executive control functions (shifting, updating, and inhibition) being proposed by Miyake et al. (2000) explained method variance in the IAT. For IAT scores calculated with the now standardly used D-algorithm [developed by Greenwald et al. (2003)], the authors found individual differences in task-switching ability to explain method-specific variance in the IAT resulting in decreasing IAT effects with increasing switching ability. Compared to the conventional algorithm (Greenwald et al., 1998), however, the D-algorithm was proven to substantially reduce (but not to eliminate) the amount of unwanted method-variance in the IAT effect in this study (see also Back et al., 2005).

Similar to the role of individual differences in executive functions, it is widely accepted that interindividual variability in personality and temperamental traits strongly contribute to variation in human behavior. Several lines of evidence demonstrate a relationship between personality and executive control functions suggesting that personality may be a source of method variance in the IAT. For example, converging evidence shows that negative emotionality (e.g., trait anxiety) can impair set shifting behavior (Eysenck et al., 2006), working memory updating (Gray et al., 2005), and inhibitory control (Wood et al., 2001). Accordingly, in their attentional control theory (ACT), Eysenck et al. (2007) proposed that anxiety can disrupt the balance between two attentional systems proposed by Corbetta and Shulman (2002), by decreasing the influence of the goal-directed attentional system (top-down control), but increasing the impact of the stimulus-driven attentional system (bottom-up control). According to ACT, this, in turn, results in less attentional control, higher distractibility by task-irrelevant stimuli and reduced ability to inhibit prepotent responses, especially in tasks with high demands on the central executive (Eysenck et al., 2007; p. 348).

In addition, a large body of evidence demonstrates that individuals high vs. low in anxiety show preferential or facilitated processing for negative and threat-related stimuli, which is associated with faster responding compared to neutral or positive ones (e.g., MacLeod and Rutherford, 1992; Mogg and Bradley, 1998). Given that personality IATs often implement category labels and stimuli of affective valence and that this valence might trigger recoding strategies contributing to the IAT in an unwanted manner (see Schnabel et al., 2006), negative emotionality also appears to be a relevant factor for unwanted variance in the IAT that relates to the features of the IAT material.

Further, a growing number of studies suggest an association between *extraversion* and the amount of top-down control in task performance. This relationship has already been suggested in Eysenck's arousal theory of extraversion (1967). According to this theory, extraverts possess chronically lower levels of tonic activity in the ascending reticular activation system and thus need to have more cortical stimulation to attain their optimal level of arousal relative to introverts. Consistently, extraverts as compared to introverts were found to show better task performance under conditions of higher cognitive load (e.g., faster responses in the 2- and 3-back condition of an n-back working memory task). They also performed better in multitasking contexts (Lieberman and Rosenthal, 2001). Similarly, extraverts were found to better divide attention between competing tasks (Szymura and Necka, 1998), to be less distracted by task irrelevant stimuli such as background music (Furnham and Strbac, 2002), and to have better short-term memory capacity (e.g., Howarth and Eysenck, 1968). In contrast, introverts have been shown to be superior in long-term memory (e.g., Howarth and Eysenck, 1968) and vigilance tasks (e.g., Bakan, 1959; Koelega, 1992).

Based on this evidence, it appears plausible that individuals higher in extraversion are better able to handle IAT task demands. Specifically, for each IAT trial, individuals must decide whether the stimulus belongs to one of the target- or to one of the attribute concepts and whether the item requires responding with the left or the right response key. Thus, the IAT can be compared with a multi-task setting where the ability to divide attention, larger short-term memory capacity (i.e., the maintenance of rules and categories), and lower sensitivity to distraction are of advantage. Given that such cognitive control processes are more likely to be involved in the incompatible block where task demand is increased, individual differences in extraversion may affect the IAT blocks asymmetrically, possibly leading to unwanted method variance in the IAT effect.

Taken together, personality differences may affect the IAT effect not only by construct-related, but also by confounding variance due to personality-related modulation of cognitive functions or by a particular mode of processing (e.g., facilitated processing of negative vs. neutral/positive stimuli; differential processing under low vs. high cognitive-load). To address this question, the Big-Five personality dimensions were assessed via self-report, peer-report and IAT and structural equation modeling (SEM) was used to separate trait-specific from unwanted variance.

## Materials and methods

### Participants and procedure

The sample consisted of 270 students who gave written informed consent prior to the beginning of the study and who received either monetary compensation or course credit for participation. Participants performed the “task-switching ability” IAT (TSA-IAT, Back et al., 2005) and measures of need for cognition that are reported elsewhere (Fleischhauer et al., 2013). After a 15 min break providing sufficient time for recovery, the Big-Five IATs and finally the NEO-FFI questionnaire were completed. Moreover, all participants nominated two persons who were of about the same age, who knew them well and agreed to rate their friends' personality (peer-report). Five participants were excluded from the sample because of missing IAT data or because their mean error rate in IAT performance exceeded 30%. For 61 participants, no peer-report of personality (which was required to estimate the latent personality variables in the SEMs) could be obtained. Hence, the final sample comprised 204 participants (95 males, age mean ± SD 23.1 ± 4.0 years, range 18–42 years).

### Measures

#### Indirect measures

The implicit Big-Five dimensions were measured with IAT subtests developed by Schmukle et al. (2008), which were presented in the following order (the IATs contained the target concepts “Self” vs. “Others” and the attribute concepts depicted in parentheses): (1) E_imp_ (Extraversion vs. Introversion), (2) N_imp_ (Anxiety vs. Calmness), (3) O_imp_ (Openness vs. Narrow-Mindedness), (4) A_imp_ (Agreeableness vs. Disagreeableness), (5) C_imp_ (Conscientiousness vs. Carelessness). In the TSA-IAT (Back et al., 2005), participants sort stimuli from letter and number categories (e.g., *N*, 5) as well as from word and calculation categories (e.g., shirt, 7−4 = 3). The IAT data were aggregated according to the D_1_ algorithm (see Greenwald et al., 2003). For the personality IATs, mean reaction time (RT) of the block combining “Self” with the attribute indicating high values in the respective trait (e.g., “Openness”) was subtracted from RT in the block combining “Self” with the opposite attribute (e.g., Narrow-Mindedness). Thus, positive IAT effects indicate a tendency toward high values in implicit Openness whereas negative IAT effects indicate a tendency toward low values in implicit Openness. Internal consistencies of the IATs (see Table 1) were computed as Spearman-Brown corrected split-half correlations based on two subsets of alternating trial-pairs in the combined blocks (i.e., subset 1 containing the trials 1, 2, 5, 6, […] and subset 2 including the trials 3, 4, 7, 8, […]).

**Table 1:** **Means, standard deviations, reliabilities, and intercorrelations of all measures**.

			**SR**					**PR**					**IAT**					
	***M***	***SD***	**N**	**E**	**O**	**A**	**C**	**N**	**E**	**O**	**A**	**C**	**N**	**E**	**O**	**A**	**C**	**TSA**
**SR**																		
N	22.2	8.9	0.86															
E	29.7	6.8	**−0.44**	0.82														
O	32.8	6.1	−0.05	**0.14**	0.69													
A	31.3	6.5	**−0.18**	**0.38**	0.11	0.79												
C	30.5	7.5	−0.11	**0.17**	**−0.15**	0.11	0.86											
**PR**																		
N	21.7	8.4	**0.44**	**−0.27**	0.06	−0.04	−0.02	0.86										
E	29.1	7.1	**−0.22**	**0.51**	−0.04	**0.28**	0.09	**−0.42**	0.82									
O	30.3	6.3	0.03	0.02	0.**56**	0.05	**−0.22**	0.02	0.12	0.71								
A	31.4	7.1	−0.03	**0.26**	−0.04	**0.52**	−0.01	**−0.24**	**0.44**	0.10	0.80							
C	33.4	8.3	0.07	−0.09	**−0.14**	−0.01	**0.52**	−0.05	0.09	−0.05	0.13	0.88						
**IAT**																		
N	−0.20	0.26	**0.16**	−0.11	0.06	0.05	−0.05	0.13	−0.06	0.01	−0.01	0.01	0.56					
E	0.08	0.34	**−0.24**	**0.29**	−0.03	0.11	0.01	**−0.26**	**0.22**	−0.05	0.08	−0.11	−0.13	0.76				
O	0.23	0.25	−0.11	0.08	0.01	−0.02	0.09	**−0.19**	0.14	−0.08	0.03	−0.06	**−0.16**	**0.23**	0.61			
A	0.34	0.26	0.05	−0.05	0.03	0.01	**0.15**	**0.15**	−0.04	0.11	−0.03	−0.03	−0.12	0.08	**0.23**	0.63		
C	0.27	0.25	0.03	0.01	−0.04	−0.05	**0.16**	−0.02	0.02	−0.09	−0.03	−0.01	−0.10	0.09	**0.29**	**0.25**	0.63	
TSA	1.01	0.20	−0.13	0.09	0.08	−0.02	−0.07	−0.09	0.06	−0.01	−0.02	−0.07	**−0.17**	**0.15**	0.11	0.06	**0.15**	0.69

SR, self-report; PR, peer-report; IAT, Implicit Association Test; N, neuroticism; E, extraversion; O, openness; A, agreeableness; C, conscientiousness; TSA, content-unrelated control IAT; reliabilities of self- and peer-reports (Cronbach's alpha) and of IATs (Spearman-Brown corrected split-half correlation) are shown in the diagonal, significant correlations (p < 0.05) are depicted in bold.

#### Direct measures

The explicit Big-Five factors neuroticism (N_exp_), extraversion (E_exp_), openness (O_exp_), agreeableness (A_exp_), and conscientiousness (C_exp_) were assessed by the German NEO-Five-Factor-Inventory (NEO-FFI, Borkenau and Ostendorf, 1993). Additionally, peer-reports of the NEO-FFI were collected. Peer-reports have been shown to be a valuable supplemental source of information about an individual's personality potentially increasing the validity of personality assessment (see e.g., Kolar et al., 1996; Vazire, 2006; Vazire and Mehl, 2008). The nominated peers were contacted per email by the researchers and asked to answer the items of the NEO-FFI (in the third-person form) via online questionnaire. For 204 participants, at least one peer-report was available and considered for SEM. When both peer-reports were available (*N* = 157), the friend was chosen who knew the participant longest and best. The average number of years they knew each other was 7.2 (*SD* = 7.1), and 92.2% of the informants reported to know the rated person “well” (35.3%) or “very well” (56.9%).

### Statistical analyses

To investigate effects of personality on unwanted IAT variance, SEM with maximum likelihood estimation was performed using lavaan (Rosseel, 2012) and R 2.15.1 (R Core Team, 2012). Model fit was assessed by Satorra-Bentler adjusted chi-square test statistics and the following descriptive fit indices (see Hu and Bentler, 1998, 1999; Schermelleh-Engel et al., 2003): Root Mean Square Error of Approximation (RMSEA) with its associated 90% confidence interval (CI), Standardized Root Mean Square Residual (SRMR), and Comparative Fit Index (CFI). The scaled chi-square difference test was used to compare the model fit of nested models (see Rosseel, 2012). Additionally, the sample-size adjusted Bayesian Information Criterion (*SABIC*; Sclove, 1987) was used as parsimony goodness of fit index with lower values indicating better model fit.

#### Analysis 1: effects of personality on method variance in the IAT

**Figure 2:**
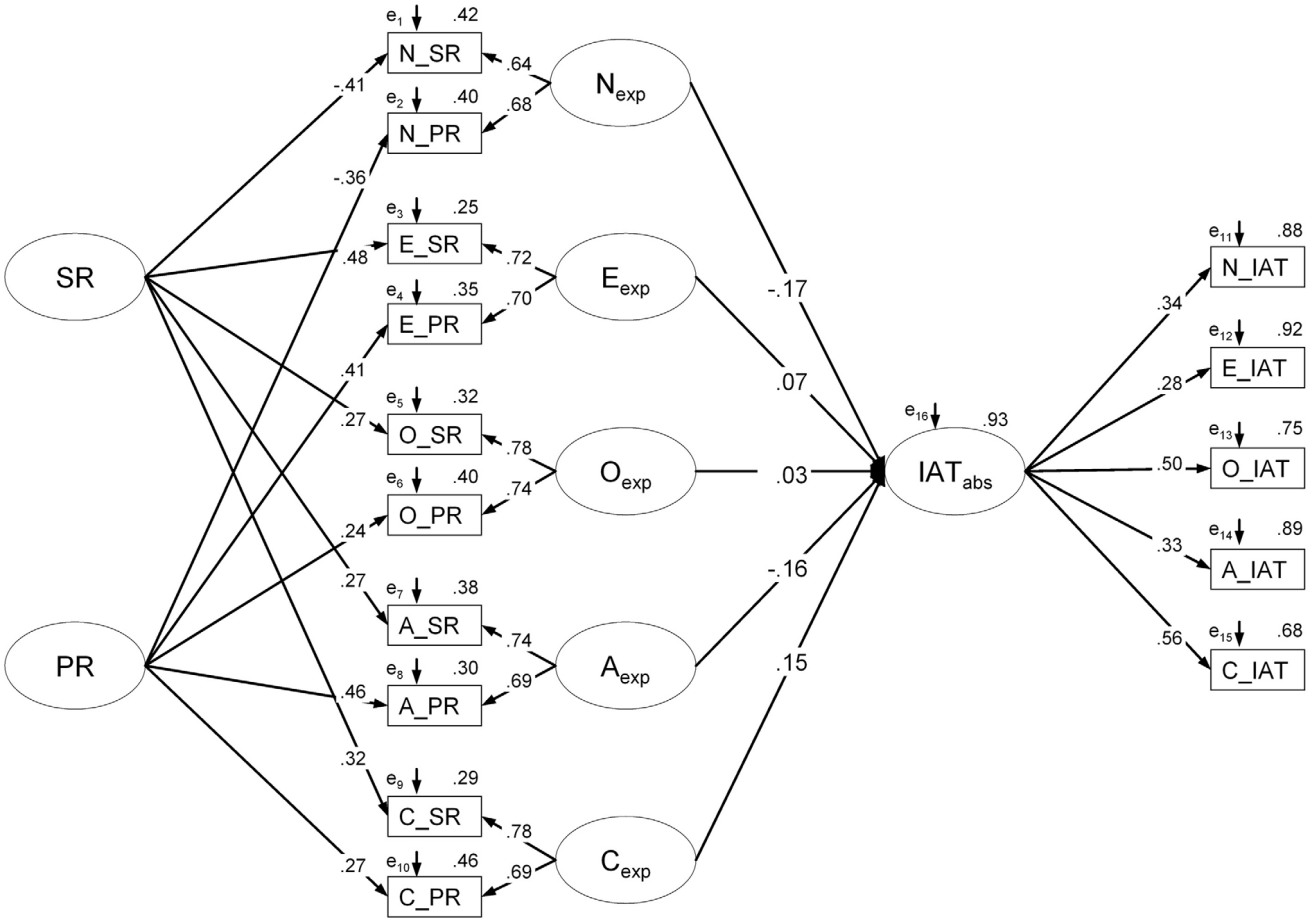
**Structural equation model with the IAT factor (IAT_abs_) explaining variance in absolute IAT scores**. Parameter estimates are fully standardized. Note that for reasons of readability, intercorrelations among the latent personality factors are not depicted. SR, self-report; PR, peer-report; IAT, Implicit Association Test; N, neuroticism; E, extraversion; O, openness; A, agreeableness; C, conscientiousness.

In Analysis 1, according to the literature (e.g., Back et al., 2005; Klauer et al., 2010), “unwanted variance” in the IAT was defined as method-specific variance. As outlined in the Introduction, method-specific variance is mainly attributable to the IAT's block design. That is, because of the higher amount of task demand in the incompatible relative to the compatible block, individual differences in, for example, task-switching ability (Klauer et al., 2010) may affect the two IAT blocks asymmetrically, thereby potentially biasing the IAT effect. The more cognitive control is exerted in the incompatible block, the smaller RT differences between both blocks (and thus IAT effects) should be (see e.g., De Houwer, 2001; Mierke and Klauer, 2001, 2003; Rothermund and Wentura, 2001, 2004). To measure personality-related effects on this method-specific variance, first, a measurement model was constructed as follows: Latent *explicit personality* factors were indicated by self- and peer-report because a multi-informant approach allows separating trait from error variance leading to more valid factors of explicit personality. Similarly to Back et al. (2005) and Klauer et al. (2010), the personality IAT scores calculated according to the D_1_ algorithm (Greenwald et al., 2003) were transformed into absolute values and used as indicators for the latent factor of *IAT method variance* (IAT_abs_). The absolute values ignore the direction of the IAT effect (i.e., whether an individual is rather anxious or not) but consider the size of the IAT effect (whether the IAT effect is close to or far from zero). Second, we tested whether additional regressions of the IAT factor (IAT_abs_) on the latent personality factors substantially improved model fit, which would indicate that personality influences method-specific variance in the IAT (see Figure 2, see Supplementary Material for the R code and the covariance matrix).

#### Analysis 2: effects of personality on unwanted IAT variance in a broader sense

In a *second analysis*, “unwanted variance” was defined in a broader sense. That is, the personality IAT scores calculated by the D_1_ algorithm (Greenwald et al., 2003) were used in their raw format (i.e., taking into account the direction of IAT effects not only their size) and unwanted variance was defined as systematic variance that cannot be explained by the implicit trait factors. Such unwanted variance components, for example, may result from individual differences in the processing of task-irrelevant features (see De Houwer, 2009) such as the valence of categories/stimuli, which may promote recoding strategies (see e.g., Govan and Williams, 2004; Bluemke and Friese, 2006; Schnabel et al., 2006) that, in turn, may bias the IAT effect in a more negative or more positive direction. Thus, this analysis would cover aspects of variance that are not reflected by IAT_abs_ as considered in Analysis 1.

Accordingly, first, a measurement model was constructed. Based on findings suggesting that implicit and explicit representations of the self-concept can best be conceived as distinct, but slightly correlated constructs (e.g., Nosek and Smyth, 2007; see also Hofmann et al., 2005; Back et al., 2009), *latent explicit* and *latent implicit* personality factors were estimated. Self- and peer-report served as indicators for the latent explicit trait factors. Latent implicit personality factors were indicated by the two IAT subsets used for computing split half reliability (see above). Note that the intercorrelations among the latent explicit personality factors as well as between the explicit and implicit trait factors were initially freely estimated, but constrained to zero in a stepwise fashion if their respective correlation amounted to *r* < 0.05. Additionally, for each of the three types of measures (self-report, peer-report, IAT), a latent measurement factor was estimated, reflecting the systematic shared variance that is not explained by the latent trait factors.

**Figure 3:**
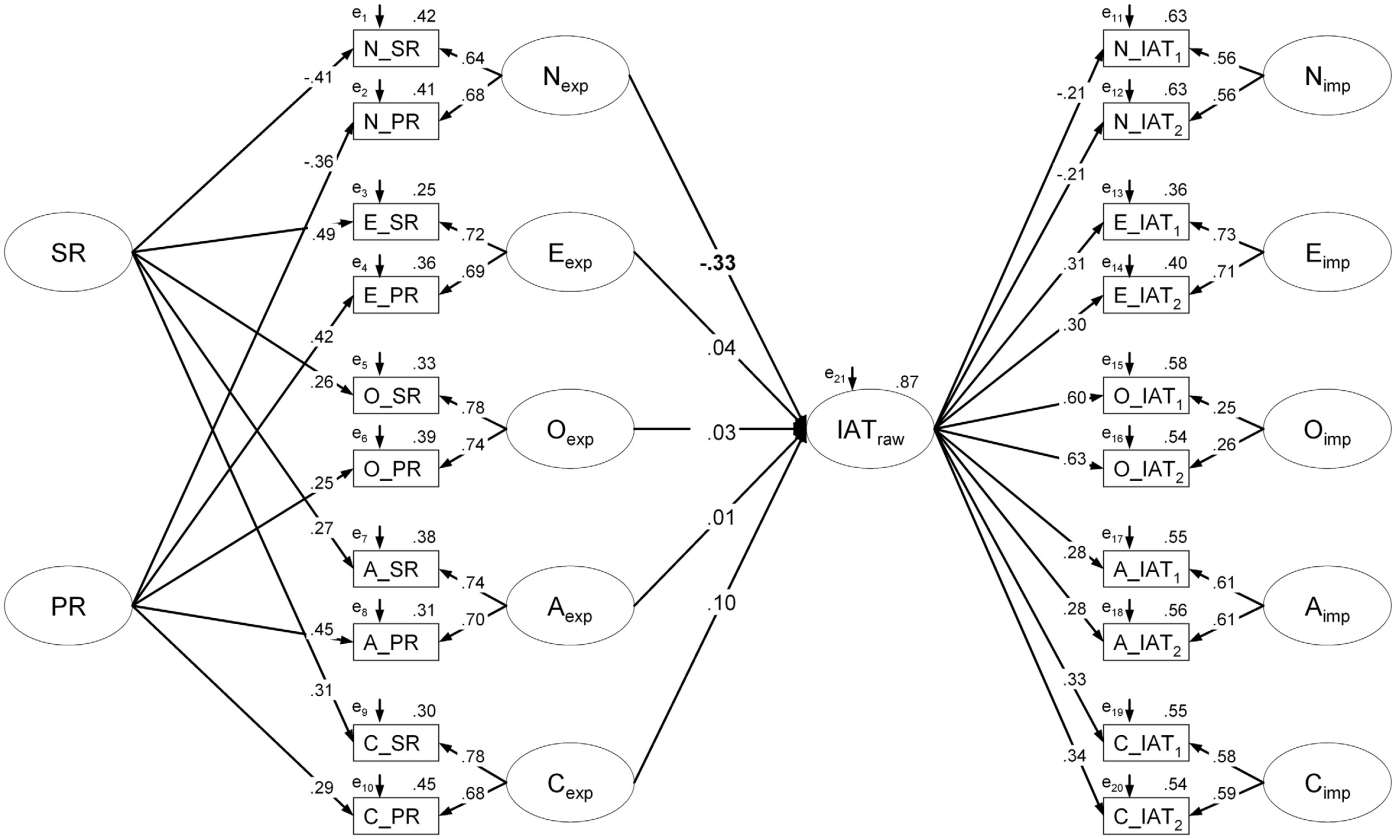
**Structural equation model with the IAT factor (IAT_raw_) explaining variance in raw IAT scores**. Parameter estimates are fully standardized. Note that for reasons of readability, intercorrelations among the latent personality factors are not depicted. Significant regression weights (*p* < 0.05) are depicted in bold. SR, self-report; PR, peer-report; IAT_1/2_, Implicit Association Test subtest 1/2; N, neuroticism; E, extraversion; O, openness; A, agreeableness; C, conscientiousness.

Second, the latent IAT measurement factor (IAT_raw_) was regressed on the latent explicit personality factors to estimate the influence of personality on unwanted IAT variance (see Figure 3, see Supplementary Material for the R code and the covariance matrix). Note that we restricted the regressions to the explicit personality factors as the IAT factor and the implicit personality factors explain variance in the same indicators, and thus covariation between those factors is difficult to interpret.

#### Relationship between the IAT factors and the content-unrelated TSA-IAT

Finally, by correlation analysis, the shared variance of the two IAT factors (IAT_abs_ and IAT_raw_) was examined. Moreover, intercorrelations with the content-unrelated TSA-IAT were estimated to gain further evidence for the amount of unwanted variance in IAT_abs_ and IAT_raw_.

## Results

### Descriptive statistics and intercorrelations

Descriptive statistics, reliabilities, and intercorrelations for the personality measures and the TSA-IAT are depicted in Table 1. The pattern of intercorrelation between personality dimensions largely replicates the results of studies using direct and indirect Big-Five measures (Schmukle et al., 2008; Back et al., 2009).

### Effects of personality on unwanted variance in the IAT

#### *Analysis 1* (IAT_abs_)

As can be seen in Table 2, the model that was based on the absolute scores of the IAT effects considering unwanted IAT variance in the sense of more or less large IAT effects (i.e., systematic method variance) fitted the data well. However, when regressions of IAT_abs_ on the five explicit personality factors were additionally included, model fit did not substantially change [Δ χ^2^(5) = 4.30, *p* = 0.507] and none of the five regressions reached significance (all *ps* > 0.20) indicating that personality had no substantial influence on IAT_abs_ (see Figure 2). However, in previous research (Back et al., 2005; Klauer et al., 2010), it has been shown that the D-measure (Greenwald et al., 2003) compared to the conventional scoring algorithm (Greenwald et al., 1998) may reduce the amount of method variance in the IAT effect. That is, the null-relationship between the trait factors and IAT_abs_ might not only refer to the fact that personality does not modulate whether individuals have smaller or larger IAT effects. It might also suggest that that the D-measure effectively controls for unwanted effects of personality on cognitive functioning during the IAT and that effects of personality would have occurred if the conventional scoring algorithm had been used. To disentangle which of the two possibilities is most likely, we repeated the modeling using the absolute values of the conventionally scored IAT effects. Again, however, none of the trait factors significantly predicted IAT_abs_ (all *ps* > 0.20) and the model fit of the measurement model [χ^2^(80) = 72.94, *p* = 0.699, *CFI* = 1.00, *RMSEA* = 0.00 (CI_90%_: 00, 0.03), *SRMR* = 0.05, SA*BIC* = 11219.67] and of the structural model [χ^2^(75) = 69.53, *p* = 0.657, *CFI* = 1.00, *RMSEA* = 0.00 (CI_90%_: 00, 0.03), *SRMR* = 0.04, SA*BIC* = 11227.19] did not significantly differ [Δ χ^2^(5) = 3.346, *p* = 0.647].

**Table 2:** **Model fit statistics**.

	***df***	**χ *^2^***	***p***	***RMSEA***	***CI*_90%_**	***SRMR***	***CFI***	***SA BIC***
**(A) ANALYSIS 1 (IAT_abs_)**								
Measurement model	80	77.34	0.563	0.00	0–0.04	0.05	1	13018.61
Structural model: *N*_exp_, *E*_exp_, *O*_exp_, *A*_exp_, *C*_exp_	75	73.09	0.541	0.00	0–0.04	0.05	1	13024.55
**(B) ANALYSIS 2 (IAT_raw_)**								
Measurement model	157	180.70	0.095	0.03	0–0.04	0.07	0.97	14340.15
Structural models: *N*_exp_, *E*_exp_, *O*_exp_, *A*_exp_, *C*_exp_	152	172.70	0.120	0.03	0–0.04	0.06	0.97	14342.71
*N*_exp_ *only*	156	173.44	0.161	0.02	0–0.04	0.06	0.98	14334.94

Model fit was assessed by Satorra-Bentler adjusted chi-square test statistics and RMSEA, root mean square error of approximation; CI_90%_, confidence interval of RMSEA; SRMR, standardized root mean square residual; CFI, comparative fit index; and SABIC, sample-size adjusted Bayesian Information Criterion.

#### *Analysis 2* (IAT_raw_)

In a second analysis (where unwanted variance was considered in a broader sense), the measurement model assuming a latent factor for each measurement (self-report, peer-report, and IAT) and latent explicit and implicit personality factors fitted the data well (see Table 2). Model fit was also excellent when the regressions of IAT_raw_ on the latent factors of the explicit Big-Five personality dimensions were additionally included (see Table 2 and Figure 3). Considering the regression paths, N_exp_ explained variance in IAT_raw_ (β_Nexp_ = −0.33, *z* = −2.27, *p* = 0.023) whereas the regression weights of the other predictors did not reach significance (all *ps* > 0.30). Therefore, regression path of IAT_raw_ on E_exp_, O_exp_, A_exp_, and C_exp_ were eliminated from the SEM. As indicated by likelihood ratio test [Δ χ^2^(4) = 0.80, *p* = 0.938], this more constrained model fitted the data equally well than the less constrained SEM (for fit indices, see Table 2) and is thus to be preferred. A likelihood ratio test comparing the final SEM with the measurement model further shows that considering N_exp_ as predictor of IAT_raw_ substantially improved model fit [Δ χ^2^(1) = 7.11, *p* = 0.008] and accounted for a substantial amount of variance [*R*^2^(*IAT*_raw_) = 11.3%]. Subsequent correlation analyses between IAT_raw_ and the two indicators of latent N_exp_ further suggest that the effect results from both, self-reported neuroticism (*r* = −0.14, *p* = 0.056) and (even more pronounced) peer-reported neuroticism (*r* = −0.18, *p* = 0.012).

### Correlations of IAT factors and TSA-IAT

Both IAT factors were highly associated (*r* = 0.83, *p* < 0.001). Moreover, they showed similar correlations with the content-unrelated and neutral TSA-IAT (IAT_abs_: *r* = 0.177, *p* = 0.011; IAT_raw_: *r* = 0.194, *p* = 0.005) indicating shared unwanted variance. Subsequent partial correlation analyses revealed that when the variance of the respective other IAT factor was eliminated (e.g., IAT_abs_ out of IAT_raw_), correlation with the TSA-IAT were reduced to insignificance (TSA and IAT_abs_: *r* = 0.029, *p* = 0.685; TSA and IAT_raw_: *r* = 0.087, *p* = 0.219) suggesting that the correlation with the TSA-IAT, reported above, was due to the shared variance of both IAT factors. That is, IAT_raw_ might also contain variance components that refer to the size of the IAT effects resulting from the block design of the IAT (i.e., due to different task demands between the compatible and incompatible blocks).

## Discussion

Recent research has shown that the IAT may be affected by confounding factors. Given their large contribution to behavioral variation, the aim of the present study was to examine the role of personality differences in unwanted variance in the IAT. The Big-Five personality dimensions were assessed with multiple measures (self-report, peer-report, and IAT). In a first analysis, we examined effects of personality on unwanted variance that is directly related to the IAT procedure (i.e., method variance). Such variance components have been shown to mainly result from the block design of the IAT, that is, the higher task demands in the incompatible block compared to the compatible block (e.g., Mierke and Klauer, 2003; Rothermund and Wentura, 2004; De Houwer et al., 2005). Consequently, individual differences in cognitive skills (e.g., Klauer et al., 2010) have been shown to contribute to the IAT effect with smaller IAT effects for individuals who exert a higher amount of cognitive skills in the more demanding incompatible block. To assess method variance, IATs were scored in terms of absolute values (i.e., its distance from zero, ignoring the sign; see e.g., Back et al., 2005; Klauer et al., 2007).

Given the literature, we expected neuroticism and extraversion to explain method variance in the IAT. Specifically, neuroticism has been related to impaired performance in cognitive tasks (Wood et al., 2001; Gray et al., 2005; Eysenck et al., 2006), whereas extraversion has been observed to be positively associated with performance under higher cognitive load and in multitasking contexts (e.g., Lieberman and Rosenthal, 2001); settings that might be comparable to the more difficult incompatible block of the IAT. However, there was no evidence for confounding effects of N_exp_ and E_exp_ or other personality factors of the Big Five on method variance as reflected in IAT_abs_. This was not only the case when the IAT scores were calculated according to the D_1_ algorithm (Greenwald et al., 2003) that might have effectively controlled for unwanted effects of personality during the IAT (see also Back et al., 2005; Klauer et al., 2010). The null relationship between personality and IAT_abs_ was also observed for the conventional scoring algorithm (Greenwald et al., 1998). Thus, we can only speculate why the expected impact of neuroticism and extraversion on the size of the IAT effects (as reflected in IAT_abs_) was not observed in this sample. With respect to extraversion, for example, in the study of Lieberman and Rosenthal (2001), differences in task performance particularly occurred when the task was the secondary but not the primary task in the multi-tasking context and when a working memory task was used. Thus, the null relationship between extraversion and IAT_abs_ might be explained by the fact that in the demanding incompatible block of the IAT, both, the target and attribute categorization task are of similar priority and that working memory performance appears to be less relevant for IAT performance relative to task-switching (Klauer et al., 2010). All in all, with respect to our sample, the results appear promising as they may suggest that personality does not substantially affect cognitive control processes that are triggered by the two differently demanding IAT blocks and that are reflected in more or less large IAT effects.

Additionally, we considered unwanted variance in a broader sense: By means of SEM, systematic IAT variance that was unique to the respective implicit trait (indicated by latent implicit trait factors) was separated from systematic common variance that goes beyond this trait variance (indicated by the latent IAT_raw_ factor). N_exp_ was observed to explain a substantial amount of variance (11%) in IAT_raw_. Specifically, higher scores in N_exp_ predicted lower IAT_raw_. However, the question is whether and to what extent this kind of shared variance indeed reflects something “unwanted.” With respect to the factor loadings of the raw IAT effects, it is noticeable that the neuroticism IAT contributed negatively to latent IAT_raw_ whereas all other IATs showed positive loadings. As the combination of “self” with the attribute concept “anxiety” should be perceived more negative than the combination of “self” with “extraversion,” “openness,” “agreeableness,” or “conscientiousness,” one may argue that the valence of the attribute concepts may have contributed to IAT variance relatively independent of the implicit personality factors to be measured. In this context, Schnabel et al. (2006) argued that the valence of the IAT's attribute categories may be a source of confounding in the IAT effect due to possible recoding tendencies. In their study, two IATs measuring anxiety and angriness, respectively, were presented in counterbalanced order. Interestingly, the two IATs showed substantial positive correlation when the anxiety IAT was presented first. This was discussed as being due to a larger amount of affective valence in the anxiousness IAT, which might have encouraged participants to recode the attribute categories from “anxious vs. self-confident” into “negative vs. positive” and to classify stimuli of these categories according to their valence rather than to their semantic meaning. A possible transfer of this strategic recoding to the subsequently presented angriness IAT may explain the positive correlation between both IATs. Similarly, the Big-Five IATs used in this study provide attribute category labels that contain a certain degree of affective valence. Accordingly, largest factor loading on IAT_raw_ was observed for the Openness IAT that contrasted the highly negatively valenced category “Narrow-Mindedness” with the highly positively valenced category “Openness.” Compared to the other Big-Five IATs, these attribute labels vary highly in negative and positive valence, and thus might have motivated individuals to recode the “Self and Openness left” vs. “Others and Narrow-mindedness right” categorization into a “positive left” vs. “negative right” categorization. As this common feature dichotomization (positive vs. negative) was not possible in the block where individuals had to press the left key for “Self and Narrow-Mindedness” and the right key for “Others and Openness,” the valence confound affects the two combined blocks asymmetrically and thus can contribute to the IAT effect. A larger IAT effect then should reflect not only the strength of associations between “Self” and “Openness” relative to “Self” and “Narrow-Mindedness” as intended, but also the amount of positive valence conveyed by “Openness” relative to “Narrow-Mindedness.” One may argue that due to a rather positive self-concept of individuals, recoding according to the single feature dichotomy “positive vs. negative” should typically occur in the block where “Self” (“Others”) is combined with a positively (negatively) valenced attribute category. Consequently, given the scoring procedure used (see Materials and Methods), recoding according to valence may result in a bias toward negative IAT scores (indicating low values in the respective implicit trait) when performing the neuroticism IAT (where Calmness is the positive attribute) but in a bias toward positive IAT score when performing the other four Big-Five IATs.

In this regard, there is a considerable amount of evidence suggesting that neuroticism or anxiety facilitates processing of negative and threat-related signals (e.g., MacLeod and Rutherford, 1992; Mogg and Bradley, 1998). Interestingly, Chan et al. (2007) examined effects of neuroticism on the evaluation of personality characteristics as desirable or undesirable and in the perception of emotions in facial expressions. They found individuals high in neuroticism to be faster in classifying negative/undesirable personality traits as compared to positive/desirable traits and to need more intense facial expressions to correctly classify positive emotions. Further, in a priming study, Robinson et al. (2007) showed that neuroticism was positively correlated with negative, but not with positive priming, which was discussed as reflecting “a greater spread of activation among negative thoughts […] within semantic memory” (p. 1229). Given these results, the negative relationship between N_exp_ and IAT_raw_ may be due to the fact that individuals high and low in neuroticism differ with respect to their processing of IAT conditions where “self” is combined with a negative/undesirable attribute concept relative to blocks with “self-positive” combinations. In specific, the facilitated processing of negatively valenced information may lead to a less pronounced bias toward negative IAT scores in the neuroticism IAT and toward positive IAT scores in the other four Big-Five IATs in individuals high in neuroticism compared to their low neuroticism counterparts.

Alternatively, our result pattern may suggest that individuals high in N_exp_ tend to spontaneously associate negative rather than positive traits with themselves (i.e., have a more negative implicit self-concept). Although lower self-esteem is a known correlate of neuroticism (Robins et al., 2001a,b), such variance in the IAT can be regarded as confounding as it would indicate that the IAT effect does not reflect the respective implicit Big-Five personality domain only (e.g., implicit Openness) but also reflects the ease with which one associates positive vs. negative stimuli with the self-due to self-esteem (see Schnabel et al., 2006; p. 390; see also Rudman et al., 2001).

Overall, our results suggest that the valence of the attribute concepts may have caused additional unwanted variance in the IAT effects as reflected by IAT_raw_. Because neuroticism has been found to especially facilitate the processing of negative stimuli compared to stimuli with positive valence, this might explain why neuroticism but not the other Big-Five domains was observed to significantly predict such valence confounds in the IAT. There was no personality-related association with IAT_abs_ reflecting systematic method variance. As indicated by additional correlation analyses, both IAT factors shared a large amount of variance (69%) and were significantly associated with the content-unrelated TSA-IAT suggesting that IAT_raw_ also relates to the size of IAT effects. Nevertheless, the results indicate that N_exp_ was predictive for that proportion of IAT_raw_ that is not shared with IAT_abs_. This, in turn, may illustrate the usefulness of investigating unwanted variance in a broader sense that goes beyond pure method variance and additionally considers variance components that may be due, for example, to features of the material (e.g., the valence of category labels) resulting in more or less negative or positive IAT effects.

To avoid bias due to the recoding according to the valence of attribute labels (and/or items representing these categories), these stimuli should be balanced by their affective valence. Such valence balancing might be particularly necessary with respect to personality IATs. That is, in self-esteem or attitude IATs, the self-concept targets such as “Me vs. Not Me” (e.g., Schröder-Abé et al., 2007) and attitude targets such as “Black vs. White Americans” (e.g., Greenwald et al., 1998, Experiment 3) are typically combined with attribute categories containing affective valence such as “Pleasant vs. Unpleasant.” In these IATs, however, the valence in the attribute concepts is considered necessary to evaluate the target concepts (e.g., in the self-esteem IATs, it is measured whether the self is implicitly perceived as being rather good or bad). In personality IATs, however, where the association between the self-concept and the semantic meaning instead of the valence of attribute categories are of interest, labels and stimuli should be controlled in terms of their valence (see also Perkins and Forehand, 2006).

Several potential limitations of the present research must be noted that may also suggest directions for future research. An alternative option to separate content-related from content-unrelated variance is to decompose the IAT effect into process components using diffusion modeling, as demonstrated by Klauer et al. (2007). The authors showed that particularly the process component *a* reflecting speed-accuracy settings significantly predicted method variance in the IAT whereas the drift rate *v* reflecting the speed of information accumulation was predictive for construct-related variance. To examine trait-specific effects on these components would be interesting. This was not possible in the present study as it would also have required the recording of the response latency on first erroneous responses as well as a larger number of trials than in standard IAT implementations to obtain a sufficient number of error trials necessary for modeling (see Klauer et al., 2007). However, prolonged IAT procedures may be more likely to be influenced by effects of task demand and motivation than standard IATs.

Moreover, it would be important to investigate to what extent effects of neuroticism affect the IAT's validity, for example, by moderating behavioral predictions of the IAT or explicit-implicit consistencies. In this context, one might examine effects of neuroticism in the context of attitude IATs that typically use negative and positive affective attribute categories and stimuli. Such an approach would also contribute to the question whether neuroticism exerts its effects on the processing of valenced stimuli in general (then effects would similarly occur for attitude IATs) or whether the found association between N_exp_ and IAT_raw_ results from a more negative implicit self-concept of individuals high in neuroticism (then effects would be specific for personality IATs containing differently valenced attribute categories).

In addition, the study is limited in elucidating whether the valence of the category labels or rather the valence of the exemplars (or both) have caused the association between N_exp_ and IAT_raw_. In this regard, previous research found evidence that the category names mainly determine how the stimuli are categorized (e.g., De Houwer, 2001) but that the features of the individual exemplars can substantially alter the interpretation of categories especially when the exemplar features are inconsistent to the category features (e.g., Steffens and Plewe, 2001). With respect to the Big-Five IATs used in this study (see Schmukle et al., 2008), category labels but also the individual exemplars of the two attribute concepts contained affective valence. As, however, the categories and exemplars were consistent in this feature (e.g., Openness = civilized, well-educated vs. Narrow-Mindedness = primitive, uneducated), one might argue that neuroticism has affected the perception of both. This might be addressed by experimental manipulations in future studies (see Perkins and Forehand, 2006).

In conclusion, our study aimed to investigate the role of personality in explaining unwanted variance in personality IATs. There were no personality-related effects on IAT method variance as reflected in absolute IAT scores. However, we observed explicit neuroticism to be predictive for unwanted variance in the raw IAT scores indicating shared variance components that go beyond this method-specific variance and that might be due, for example, to features of the used category labels and/or stimuli. In this context, our results suggest that the observed effect of neuroticism on IAT_raw_ may have been driven by the affective valence of the attribute categories (and stimuli) in the personality IATs and by the facilitated processing of negative compared to positive/neutral information as frequently observed for individuals high in neuroticism. Thus, category labels and the individual exemplars of the attribute concepts of personality IATs should, at best, be balanced for affective valence to avoid confounding, for example, due to recoding (see also Steffens, 2004; Schnabel et al., 2006).

### Conflict of interest statement

The authors declare that the research was conducted in the absence of any commercial or financial relationships that could be construed as a potential conflict of interest.
