# Supplementary material for: Neuroticism explains unwanted variance in Implicit Association Tests of personality: possible evidence for an affective valence confound
Source: Front Psychol. 2013 Sep 30;4:672. doi: 10.3389/fpsyg.2013.00672 (PMC3786234; doi:10.3389/fpsyg.2013.00672)
Supplement: Supplementary file 1 [file Presentation1.PDF]

SUPPLEMENTARY MATERIAL

Analysis 1_ Variance/covariance matrix

	N_SR	N_PR	E_SR	E_PR	O_SR	O_PR	A_SR	A_PR	C_SR	C_PR	N_IAT_{abs}	E_IAT_{abs}	O_IAT_{abs}	A_IAT_{abs}	C_IAT_{abs}
**N_SR**	80,405														
**N_PR**	-26,111	45,717													
**E_SR**	-2,372	5,664	36,876												
**E_PR**	-9,910	16,605	3,764	42,457											
**O_SR**	-8,165	7,971	-6,410	4,964	55,829										
**O_PR**	33,376	-14,999	4,990	-2,600	-1,410	73,028									
**A_SR**	-13,324	24,089	-2,000	13,070	3,896	-25,148	49,624								
**A_PR**	1,062	0,476	22,259	1,781	-9,871	3,145	4,562	40,562							
**C_SR**	-1,881	12,322	-1,449	23,745	0,021	-14,178	21,284	4,683	49,494						
**C_PR**	4,771	-5,328	-6,185	-0,518	32,390	-2,149	4,543	-1,107	7,499	68,704					
**N_IAT_{abs}**	-0,170	0,041	-0,111	-0,080	-0,026	-0,168	0,032	-0,016	0,024	-0,063	0,036				
**E_IAT_{abs1}**	-0,110	-0,061	0,039	0,074	-0,107	-0,127	0,054	0,111	-0,016	-0,177	0,002	0,044			
**O_IAT_{abs}**	-0,166	0,080	-0,039	-0,096	0,130	-0,298	0,177	-0,006	0,012	-0,015	0,007	0,006	0,035		
**A_IAT_{abs}**	0,123	-0,026	-0,015	0,050	0,225	0,273	-0,041	0,104	0,002	-0,007	0,004	0,006	0,006	0,045	
**C_IAT_{abs}**	-0,083	-0,004	-0,042	-0,061	0,226	0,002	0,002	-0,054	-0,167	-0,008	0,007	0,007	0,009	0,009	0,039

Analysis 1_R code

```
A1_MM <- "
#Latent variable explicit
N_expl =~ nex*SR_neon + nex*PR_neon
E_expl =~ eex*SR_neoe + eex*PR_neoe
O_expl =~ oex*SR_neoo + oex*PR_neoo
A_expl =~ aex*SR_neoa + aex*PR_neoa
C_expl =~ cex*SR_neoc + cex*PR_neoc

#method factors
Met_IAT =~ iatn*absNEON_IAT + iate*absNEOE_IAT + iato*absNEOO_IAT + iata*absNEOA_IAT + iatc*absNEOC_IAT
Met_SR =~ srn*SR_neon + sre*SR_neoe + sro*SR_neoo + sra*SR_neoa + src*SR_neoc
Met_PR =~ prn*PR_neon + pre*PR_neoe + pro*PR_neoo + pra*PR_neoa + prc*PR_neoc
#Met_IAT ~~ Met_SR + Met_PR
#Met_SR ~~ Met_PR
```

```
#SR variance
SR_neon ~~ eSRn*SR_neon
SR_neoe ~~ eSRe*SR_neoe
SR_neoo ~~ eSRo*SR_neoo
SR_neoa ~~ eSRa*SR_neoa
SR_neoc ~~ eSRc*SR_neoc

#PR variance
PR_neon ~~ ePRn*PR_neon
PR_neoe ~~ ePRe*PR_neoe
PR_neoo ~~ ePRo*PR_neoo
PR_neoa ~~ ePRa*PR_neoa
PR_neoc ~~ ePRc*PR_neoc

#IAT variance constraints
absNEON_IAT ~~ eIATn*absNEON_IAT
absNEOE_IAT ~~ eIATe*absNEOE_IAT
absNEOO_IAT ~~ eIATo*absNEOO_IAT
absNEOA_IAT ~~ eIATa*absNEOA_IAT
absNEOC_IAT ~~ eIATc*absNEOC_IAT


#Latent variable covariances
N_expl ~~ ne*E_expl + no*O_expl + na*A_expl + 0*C_expl
E_expl ~~ 0*O_expl + ea*A_expl + 0*C_expl
O_expl ~~ 0*A_expl + oc*C_expl
A_expl ~~ 0*C_expl
"

A1MM_fit <- cfa(A1_MM, std.lv=T, orthogonal=T, meanstructure=T, estimator="MLM", data=rawdata)
summary(A1MM_fit, fit.measures=T, standardized=T)

A1_ SM_allexp <- paste(c(A1_MM,"Met_IAT ~ N_expl + E_expl + O_expl + A_expl + C_expl"),collapse="\n")
A1_fitSM <- cfa(A1_ SM_allexp, std.lv=T, orthogonal=T, meanstructure=T,estimator="MLM", data=rawdata)
summary(A1_fitSM, fit.measures=T, standardized=T, rsquare=T)

anova(A1_fitSM, A1MM_fit)
```

Analysis 2_Variance/covariance matrix

	**N_SR**	**N_PR**	**E_SR**	**E_PR**	**O_SR**	**O_PR**	**A_SR**	**A_PR**	**C_SR**	**C_PR**	**N_IAT$_1$**	**N_IAT$_2$**	**E_IAT$_1$**	**E_IAT$_2$**	**O_IAT$_1$**	**O_IAT$_2$**	**A_IAT$_1$**	**A_IAT$_2$**	**C_IAT$_1$**	**C_IAT$_2$**
**N_SR**	80,405																			
**N_PR**	-26,111	45,717																		
**E_SR**	-2,372	5,664	36,876																	
**E_PR**	-9,910	16,605	3,764	42,457																
**O_SR**	-8,165	7,971	-6,410	4,964	55,829															
**O_PR**	33,376	-14,999	4,990	-2,600	-1,410	73,028														
**A_SR**	-13,324	24,089	-2,000	13,070	3,896	-25,148	49,624													
**A_PR**	1,062	0,476	22,259	1,781	-9,871	3,145	4,562	40,562												
**C_SR**	-1,881	12,322	-1,449	23,745	0,021	-14,178	21,284	4,683	49,494											
**C_PR**	4,771	-5,328	-6,185	-0,518	32,390	-2,149	4,543	-1,107	7,499	68,704										
**N_IAT$_1$**	0,510	-0,250	0,029	0,057	-0,041	0,260	-0,163	-0,081	-0,116	-0,026	0,100									
**N_IAT$_2$**	0,267	-0,097	0,159	0,129	-0,197	0,319	0,020	0,085	0,070	0,047	0,038	0,104								
**E_IAT$_1$**	-0,893	0,861	-0,033	0,326	-0,080	-0,850	0,700	-0,069	0,173	-0,277	-0,017	-0,014	0,154							
**E_IAT$_2$**	-0,677	0,541	-0,200	0,161	0,103	-0,823	0,452	-0,264	0,224	-0,362	-0,010	-0,003	0,095	0,153						
**O_IAT$_1$**	-0,196	-0,024	-0,025	-0,103	0,132	-0,440	0,076	-0,170	-0,108	-0,183	-0,006	-0,014	0,021	0,022	0,089					
**O_IAT$_2$**	-0,416	0,310	0,046	0,137	0,254	-0,465	0,441	-0,059	0,238	-0,074	-0,010	-0,021	0,028	0,014	0,039	0,089				
**A_IAT$_1$**	0,019	-0,022	0,120	0,035	0,226	0,251	-0,046	0,277	-0,064	-0,162	-0,005	-0,008	0,006	0,009	0,015	0,018	0,099			
**A_IAT$_2$**	0,199	-0,100	0,040	0,057	0,360	0,438	-0,126	0,096	-0,103	-0,043	-0,010	-0,013	0,001	0,006	0,013	0,019	0,045	0,102		
**C_IAT$_1$**	-0,014	0,048	-0,019	-0,001	0,335	0,112	-0,062	-0,154	-0,143	0,171	-0,008	-0,013	0,009	0,011	0,017	0,027	0,014	0,022	0,093	
**C_IAT$_2$**	0,162	-0,024	-0,106	-0,073	0,288	-0,140	0,125	-0,132	0,015	-0,181	-0,009	-0,001	0,004	0,010	0,010	0,020	0,012	0,021	0,041	0,085

Analysis 2_R code

```
A2_MM <- "
#Latent variable explicit
N_expl =~ nex*SR_neon + nex*PR_neon
E_expl =~ eex*SR_neoe + eex*PR_neoe
O_expl =~ oex*SR_neoo + oex*PR_neoo
A_expl =~ aex*SR_neoa + aex*PR_neoa
C_expl =~ cex*SR_neoc + cex*PR_neoc

#Latent variable implicit
N_impl =~ ni*NEON_IAT_s1 + ni*NEON_IAT_s2
E_impl =~ ei*NEOE_IAT_s1 + ei*NEOE_IAT_s2
```

```
O_impl =~ oi*NEOO_IAT_s1 + oi*NEOO_IAT_s2
A_impl =~ ai*NEOA_IAT_s1 + ai*NEOA_IAT_s2
C_impl =~ ci*NEOC_IAT_s1 + ci*NEOC_IAT_s2

#method factors
Met_IAT =~ iatn*NEON_IAT_s1 + iatn*NEON_IAT_s2 + iate*NEOE_IAT_s1 + iate*NEOE_IAT_s2 + iato*NEOO_IAT_s1 +
iato*NEOO_IAT_s2 + iata*NEOA_IAT_s1 + iata*NEOA_IAT_s2 + iatc*NEOC_IAT_s1 + iatc*NEOC_IAT_s2
Met_SR =~ srn*SR_neon + sre*SR_neoe + sro*SR_neoo + sra*SR_neoa + src*SR_neoc
Met_PR =~ prn*PR_neon + pre*PR_neoe + pro*PR_neoo + pra*PR_neoa + prc*PR_neoc

#SR variance
SR_neon ~~ eSRn*SR_neon
SR_neoe ~~ eSRe*SR_neoe
SR_neoo ~~ eSRo*SR_neoo
SR_neoa ~~ eSRa*SR_neoa
SR_neoc ~~ eSRc*SR_neoc

#PR variance
PR_neon ~~ ePRn*PR_neon
PR_neoe ~~ ePRe*PR_neoe
PR_neoo ~~ ePRo*PR_neoo
PR_neoa ~~ ePRa*PR_neoa
PR_neoc ~~ ePRc*PR_neoc

#IAT variance constraints
NEON_IAT_s1 ~~ eIATn1*NEON_IAT_s1
NEON_IAT_s2 ~~ eIATn2*NEON_IAT_s2
NEOE_IAT_s1 ~~ eIATe1*NEOE_IAT_s1
NEOE_IAT_s2 ~~ eIATe2*NEOE_IAT_s2
NEOO_IAT_s1 ~~ eIATo1*NEOO_IAT_s1
NEOO_IAT_s2 ~~ eIATo2*NEOO_IAT_s2
NEOA_IAT_s1 ~~ eIATa1*NEOA_IAT_s1
NEOA_IAT_s2 ~~ eIATa2*NEOA_IAT_s2
NEOC_IAT_s1 ~~ eIATc1*NEOC_IAT_s1
NEOC_IAT_s2 ~~ eIATc2*NEOC_IAT_s2

#Latent variable covariances
N_expl ~~ ne*E_expl + no*O_expl + na*A_expl + 0*C_expl + nn*N_impl
E_expl ~~ 0*O_expl + ea*A_expl + 0*C_expl + ee*E_impl
```

```
O_expl ~~ 0*A_expl + oc*C_expl + 0*O_impl
A_expl ~~ 0*C_expl + 0*A_impl
C_expl ~~ cc*C_impl
"

A2MM_fit <- cfa(A2_MM, std.lv=T, orthogonal=T, meanstructure=T, estimator="MLM", data=rawdata)
summary(A2MM_fit, fit.measures=T, standardized=T)

A2_ SM_allexp <- paste(c(A2_MM,"Met_IAT ~ N_expl + E_expl + O_expl + A_expl + C_expl"),collapse="\n")
A2_fitSM <- cfa(A2_ SM_allexp, std.lv=T, orthogonal=T, meanstructure=T,estimator="MLM", data=rawdata)
summary(A2_fitSM, fit.measures=T, standardized=T, rsquare=T)

A2_ SM_N <- paste(c(A2_MM,"Met_IAT ~ N_expl"),collapse="\n")
A2_fitSM2 <- cfa(A2_ SM_N, std.lv=T, orthogonal=T, meanstructure=T,estimator="MLM", data=rawdata)
summary(A2_fitSM2, fit.measures=T, standardized=T, rsquare=T

anova(A2_fitSM, A2_fitSM2)
anova(A2_fitSM2, A2MM_fit)
```
